# Alzheimer’s Disease-Like Pathology Triggered by *Porphyromonas gingivalis* in Wild Type Rats Is Serotype Dependent

**DOI:** 10.3389/fimmu.2020.588036

**Published:** 2020-11-09

**Authors:** Jaime Díaz-Zúñiga, Jamileth More, Samanta Melgar-Rodríguez, Matías Jiménez-Unión, Francisca Villalobos-Orchard, Constanza Muñoz-Manríquez, Gustavo Monasterio, José Luis Valdés, Rolando Vernal, Andrea Paula-Lima

**Affiliations:** ^1^ Periodontal Biology Laboratory, Faculty of Dentistry, Universidad de Chile, Santiago, Chile; ^2^ Centro de Investigación Clínica Avanzada (CICA), Hospital Clínico Universidad de Chile, Santiago, Chile; ^3^ Biomedical Neuroscience Institute, Faculty of Medicine, Universidad de Chile, Santiago, Chile; ^4^ Department of Neuroscience, Faculty of Medicine, Universidad de Chile, Santiago, Chile; ^5^ Faculty of Dentistry, Institute for Research in Dental Sciences, Universidad de Chile, Santiago, Chile

**Keywords:** periodontitis, Alzheimer’s disease, memory, glia, neuroinflammation

## Abstract

Periodontal disease is a disease of tooth-supporting tissues. It is a chronic disease with inflammatory nature and infectious etiology produced by a dysbiotic subgingival microbiota that colonizes the gingivodental sulcus. Among several periodontal bacteria, *Porphyromonas gingivalis* (*P. gingivalis*) highlights as a keystone pathogen. Previous reports have implied that chronic inflammatory response and measurable bone resorption are observed in young mice, even after a short period of periodontal infection with *P. gingivalis*, which has been considered as a suitable model of experimental periodontitis. Also, encapsulated *P. gingivalis* strains are more virulent than capsular-defective mutants, causing an increased immune response, augmented osteoclastic activity, and accrued alveolar bone resorption in these rodent experimental models of periodontitis. Recently, *P. gingivalis* has been associated with Alzheimer’s disease (AD) pathogenesis, either by worsening brain pathology in AD-transgenic mice or by inducing memory impairment and age-dependent neuroinflammation middle-aged wild type animals. We hypothesized here that the more virulent encapsulated *P. gingivalis* strains could trigger the appearance of brain AD-markers, neuroinflammation, and cognitive decline even in young rats subjected to a short periodontal infection exposure, due to their higher capacity of activating brain inflammatory responses. To test this hypothesis, we periodontally inoculated 4-week-old male Sprague-Dawley rats with K1, K2, or K4 *P. gingivalis* serotypes and the K1-isogenic non-encapsulated mutant (GPA), used as a control. 45-days after periodontal inoculations with *P. gingivalis* serotypes, rat´s spatial memory was evaluated for six consecutive days in the Oasis maze task. Following functional testing, the animals were sacrificed, and various tissues were removed to analyze alveolar bone resorption, cytokine production, and detect AD-specific biomarkers. Strikingly, only K1 or K2 *P. gingivalis*-infected rats displayed memory deficits, increased alveolar bone resorption, pro-inflammatory cytokine production, changes in astrocytic morphology, increased Aβ1-42 levels, and Tau hyperphosphorylation in the hippocampus. None of these effects were observed in rats infected with the non-encapsulated bacterial strains. Based on these results, we propose that the bacterial virulence factors constituted by capsular polysaccharides play a central role in activating innate immunity and inflammation in the AD-like pathology triggered by *P. gingivalis* in young rats subjected to an acute experimental infection episode.

## Introduction

Periodontitis is a chronic non-communicable disease caused by a dysbiotic subgingival microbiota (1). *Porphyromonas gingivalis (P. gingivalis*) has been identified as a keystone pathogen among several bacteria present in this dysbiotic microbiota, invoking increased secretion of pro-inflammatory cytokines, matrix metalloproteinases, and pro-bone resorptive mediators that promote periodontal tissue breakdown (2, 3). *P. gingivalis* displays a wide variety of virulence factors, which have major roles in bacterial adhesion, colonization and invasion. Among the virulence factors described for *P. gingivalis*, the most important are the gingipains, lipopolysaccharide (LPS) and capsular polysaccharides, but its antigenicity is mainly related to its extracellular capsule’s polysaccharide components (4, 5).

Several studies have evaluated the virulence of the K-antigen of *P. gingivalis* for its capacity to cause an immune response in various immune cells. Encapsulated serotypes K1 and K2 are known to induce increased production of interleukin (IL)-1β, IL-6, IL-17, interferon (IFN)-γ, and tumor necrosis factor (TNF)-α in macrophages and dendritic cells, as compared with the other serotypes and the non-encapsulated strains (6, 7). Similarly, serotypes K1 and K2 induce higher differentiation of CD4^+^ T helper type 1 (Th1) and type 17 (Th17) lymphocytes compared with the others (8, 9). Besides, the K1 serotype induces bone-resorption and activates Th1 and Th17 immune response, while mice infected with the GPA strain and sham controls do not exhibit these effects (10). Furthermore, in subjects affected by periodontitis, IgG forms reactive to the K antigen are detected, and the presence of serotypes K1 or K2 is observed more frequently in periodontal sites (11, 12).

Conversely, non-encapsulated and K4 strains—associated with periodontal health—induce an immune regulatory response predominantly (8, 9). While non-encapsulated and K4 strains generate Treg response and localized abscesses, encapsulated serotypes are resistant to phagocytosis, generate distant abscesses and sepsis (8, 9, 13). The generation of the W50 strain (serotype K1)-based isogenic capsular-deficient mutants was possible after the identification and characterization of the capsular polysaccharide (K-antigen) locus of *P. gingivalis*, which contributed to the current understanding of the importance of capsule in the induction of the host immune response (14). The analysis of these mutants indicated that the presence of capsule associates with the deregulated production of pro-inflammatory cytokines by fibroblasts and macrophages, thus further supporting the idea that encapsulation is a hallmark of the virulence heterogeneity of *P. gingivalis* (15).

*P. gingivalis* can destroy the periodontal tissues and distribute in different organs and tissues, where it can produce coronary and aortic atherogenesis, favor preterm birth, and deregulate the adequate metabolic control in diabetes mellitus-affected patients (16–20). Also, recent studies have used wild-type and transgenic mice to establish the association between *P. gingivalis* and Alzheimer’s disease (AD)-related cognitive decline, by assessing the amyloid β (Aβ) peptide accumulation and pro-inflammatory cytokines production, as well as the performance in spatial memory-dependent tasks in rodents orally infected with *P. gingivalis* (21–25).

AD is a neurodegenerative disorder that affects behavioral and cognitive functions, with an early, prominent, and progressive dysfunction in the hippocampus (26, 27). The amyloid cascade hypothesis begins with the accumulation of Aβ peptide, which induces a microglial M1 inflammatory response that activates astrocytes, triggering energy, metabolic, and oxidative imbalance that leads to hyperphosphorylation of the microtubule-associated protein Tau in neurons (28, 29). A new hypothesis proposes sporadic AD as the result of independent, yet intersecting age-related pathologies that affect the aging human brain, many of which are related to neuroinflammation (30). It is believed that over time such newly generated, divergent activated microglia could sustain a chronic type of neuroinflammation in AD. This inflammatory environment, where activated microglia and reactive-astrocytes are actively producing inflammatory mediators, enhances Aβ production; its accumulation causes Tau hyperphosphorylation, which constitutes the histopathological hallmarks of AD (31). This finding led to the “neuroinflammation hypothesis” that emphasizes chronic systemic inflammatory diseases as major risk factors for AD (27).

Among chronic systemic diseases, periodontitis is the inflammatory disease more frequent in humans. Recently, brains of patients who died due to AD revealed frequent detection of *P. gingivalis*, particularly in the fourth ventricle, hippocampus, and cerebrospinal fluid (CSF) (32–34). Interestingly, brain expression levels of the pro-inflammatory cytokines and memory of mice after 6 weeks of *P. gingivalis* oral infection in young versus middle-aged (12 months old) mice. After receiving oral gavage with a *P. gingivalis* strain of reduced virulence, only middle-aged *P. gingivalis* infected mice exhibited memory impairment and increased expression levels of the pro-inflammatory cytokines TNF-α, IL-6, and IL-1β in the brain tissues, suggesting that *P. gingivalis* effects in the brain are dependent on age (22, 23).

Interestingly, the possibility that the immune response induced in the brain might differ depending on the infecting capsular *P. gingivalis* serotype has never been exploited. Microglia are known to discriminate against different bacterial antigens, triggering the activation of divergent pathways according to the antigens recognized (35–38). Thus, it is possible that some *P. gingivalis* strains might be more virulent than others in causing Alzheimer´s like-disease. Accordingly, this study aimed to evaluate whether the capsulated strains of *P. gingivalis* display divergent capacities of inducing cognitive impairment, neuroinflammation and AD-like neuropathology in young rats subjected to a short period of infection with different serotypes of this periodontal-pathogen.

## Materials and Methods

### Animals

Sprague-Dawley young male rats (4 weeks old) were obtained from the Animal Care Facility of the Faculty of Medicine at the Universidad de Chile. Rats were housed individually in a controlled environment with a 12 h light/dark cycle at 22 ± 0.5°C, 40%–70% relative humidity, with food and water *ad libitum* except when indicated otherwise; all animals were handled daily for 2 weeks before bacterial inoculation. During the 4 weeks previous to bacterial injection, all rats in each group inhabited the same cage and were fed and hydrated from the same sources. After injecting the bacteria, they were isolated in separate filter-containing cages. The experimental protocols complied and were approved by the Bioethics Committees on Animal Research of the Faculty of Medicine (protocol #CBA 0755 FMUCH) and the Faculty of Dentistry (protocol #17085-ODO-UCH), Universidad de Chile.

### Experimental Periodontitis

Each experimental group consisted of six rats housed in separate boxes conditioned with air filters maintained under standard conditions, as described above. Experimental periodontal infections were performed under general anesthesia with 2% isoflurane. Animals received an injection into the palatal mucosa of 10^10^ CFU/ml of the *P. gingivalis* strains W50 (serotype K1), HG184 (K2), or ATCC^®^ 49417™ (K4). As controls, animals were inoculated with the non-encapsulated W50 ΔPG0116-PG0120 mutant strain (GPA) of *P. gingivalis*. A final volume of 100 μl was inoculated into the palatal gingival tissues, between the first and second molar, repeating this procedure after 7 days (i.e., each rat received two palatal injections). The volume and the CFU concentration used in the palatal injections were determined based on a previous pilot study reported by our group (10). Sham-rats that received vehicle without bacteria were used for comparisons. After 45 days, animals were subjected to the Oasis Maze task to evaluate spatial memory.

### OASIS Maze Spatial Memory Task

The Oasis maze—a dry version of the classical Morris water maze memory task—was used to evaluate the hippocampal-dependent spatial memory of rats, as previously described (39–41). Animal behavior was recorded with a video camera installed in the zenithal position, and video recordings were analyzed using the Virtual Dub software (Microsoft Windows, Microsoft Corporation, Redmond, WA). The position of animals was tracked, and navigation was reconstructed and analyzed with the Matlab^®^ software (MathWorks, Cleve Moler, MA, USA). This model was chosen to replace the classical Morris Water Maze (42) to avoid oral dysbiosis by colonizing bacteria resident in the water used in the maze.

### Euthanasia and Tissue Sampling

After concluding the Oasis maze task, rats were euthanized by cervical dislocation following the American Veterinary Medical Association recommendations. Samples of maxillae, CSF, and hippocampus were immediately obtained. Extraction of CSF and blood (by cardiac puncture with 5 ml hypodermic syringe) was performed according to standard protocols (43). Blood was maintained at 37°C/2 h and at 4°C/30 min for serum isolation. Brains were removed and homogenized. Samples were centrifuged at 10.000 x *g* for 5 min at 4°C; the supernatant was recovered, its protein concentration was measured in a spectrophotometer (Bio-Tek, Winooski, VT, USA), and aliquots of 200 μl were stored at -80°C until further analysis. For histochemical analysis, brains were fixed after finishing the Oasis maze task according to published protocols (39, 40).

### Bone Destruction Quantification

To determine the pathogenicity of *P. gingivalis*, we evaluated morphological changes in the alveolar bone. The maxillary alveolar bone was scanned by µCT (Bruker microCT, SkyScan 1278; Bruker, Kontich, Belgium), and 3D image reconstructions were used to quantify bone resorption in the maxillae of rats as previously described (10, 44). The Nrecon software (Bruker, Kontich, Belgium) was used to perform the morphometric analysis, executing a linear measurement from the cementum-enamel junction (CEJ) to the alveolar bone-crest (ABC) on the 1^st^, 2^nd^, and 3^rd^ left and right upper molars.

### Immunofluorescence Analysis

For immunofluorescence analysis, 1 h after the last test session, adult rats were transcardially perfused with 300 ml of saline flush and 300 ml of 4% paraformaldehyde in 0.1 M PBS, pH 7.4. After perfusion, brains were removed, postfixed in 4% paraformaldehyde for 2 h at room temperature, and incubated for 72 h at 4°C in a solution containing 30% sucrose, 0.002% sodium azide for cryopreservation, as previously described (39). Afterward, brains were cut in the coronal plane with a sliding frozen microtome at -30°C. Free-floating 40 µm thickness sections were immersed in a blocking solution (PBS containing 0.25% Triton X-100 plus 3% donkey) serum for 2 h at room temperature. Sections were incubated overnight at 4°C with blocking solution containing primary antibodies to visualize neurons (anti-TubIII, Abcam, Ab18207), astrocytes (anti-GFAP, Abcam, ab10062), and the cytoskeleton-associated protein Tau, in its phosphorylated (anti-phosphoTau, Phospho S404, Abcam, ab92676) and total (anti-Tau, Abcam, ab64193) forms. Sections were washed three times for 5 min with PBS and incubated for 2 h with secondary fluorescent anti-mouse, goat or rabbit antibodies (Alexa Fluor^®^ 488, AlexaFluor^®^ 594 or Alexa Fluor^®^ 647, Abcam US). Nuclei were stained with Hoechst (1:10,000, Sigma, St Louis, MI, USA). Z-stack of 1.5 μm sections were captured from the hippocampus in a confocal microscope (Nikon C2+, Melville, NY.). Fluorescence intensity was measured with the confocal microscope NIS-Elements software viewer 4.0 (Nikon, Melville, NY) and with ImageJ free viewer software (National Institutes of Health, MD, USA; https://imagej.nih.gov/ij/). Sholl analysis was performed to evaluate changes in neuronal morphology. To this aim, the neuronal nucleus is located in the center of the measurement, and the software establishes concentric circles at a distance of 10 μm and calculates the number of projections that intersect for each radius and the maximum distance it reaches the most extensive projection of the cell. For the quantification of different molecules, the brains of sham rats were used as control.

### Cytokines and Aβ_1-42_ Peptide Measurements

The levels of IL-1β, IL-4, IL-6, IL-10, TNF-α, IFN-γ, in hippocampal homogenates, serum and CSF samples, and the levels of Aβ_1-42_ peptide in hippocampal homogenates and CSF were quantified using ELISA assays (R&D Systems, Minneapolis, USA or Invitrogen, Thermo Fisher Scientific, MA, USA), by evaluating the absorbance at 450 and 560 nm in an automated spectrophotometer (Bio-Tek, Winooski, VT, USA), as previously described (37). The Aβ_1-42_ peptide ELISA assay detects and quantifies mouse and rat Aβ_1-42_, both natural and synthetic. For the quantification of the different molecules, each animal was considered individually. No sample was held as a pool.

### Malondialdehyde Quantification

We used the thiobarbituric acid reactive substances (TBARS) method to measure lipid peroxidation in the hippocampus of the rats, following the manufacturer’s recommendations (Invitrogen, Thermo Fisher Scientific, MA, USA). In brief, 300 μl of hippocampus homogenates were treated with 0.6 N trichloroacetic acid and clarified by precipitating the interfering proteins. Thus, the absorbance of all samples was measured at 450 nm and 560 nm using a plate spectrophotometer (Bio-Tek, Winooski, VT, USA), as indicated by the manufacturer. This assay recognizes oxidized lipids by a reaction between malondialdehyde (MDA) and thiobarbituric acid in the presence of heat. The reaction produces TBARS, which are detected and quantified by a spectrophotometer and compared with a standard curve.

### *Porphyromonas gingivalis* Detection

The presence of *P. gingivalis* was determined by quantifying by qPCR the contents of the 16S rRNA subunit and the *RgpA* and *Kgp* genes. Total DNA was purified following the manufacturer’s instructions (FavorPrep™ Tissue Genomic DNA extraction Mini Kit, Favorgen Biotech Corp.). Briefly, samples were incubated in 200 μl FATG1 buffer and 20 μl of proteinase K and incubated at 60°C. After tissue elution, samples were incubated with 4 μl of RNAase A for 2 min, followed by incubation with 200 μl of FATG1 buffer at 70°C for 1 h, and then 200 μl of 100% ethanol were added. Each sample was inserted in a collection tube and centrifuged for 1 min at 18,000 x *g* and then washed with 400 μl of FATG buffer. Next, 2 washes were performed: 400 μl of W1 buffer and 1 min of centrifugation at 18,000 x *g* and 750 μl of wash buffer followed by 1 min of centrifugation at 18,000 x *g*. Finally, the column was incubated with 200 μl of elution buffer (pH 7.5-9.0) for 3 min and centrifuged at maximum speed for 2 min to elute the total DNA. The samples were quantified using a spectrophotometer (Bio-Tek). Genes were quantified from 50 ng of DNA by qPCR, using specific primers (**Table 1**), a KAPA SYBR^®^ FAST qPCR kit (Kapa Biosystems, MA, USA) according to the manufacturer’s instructions and using equipment from StepOne Plus. As negative controls, no-template control (NTC) and a no-amplification control omitting DNA polymerase were performed, and samples were analyzed in triplicates in each experiment.

**Table 1:** Primers used in the detection of *P. gingivalis* by qPCR.

Target	*Forward primer*	*Reverse primer*
**16S**	*5’gcgctcaacgttcagcc3’*	*5’cacgaattccgcctgc3’*
**RgpA**	*5’agtgagcgaaacttcggagc3’*	*5’ggtatcactgggtataacctgtcc3’*
**Kgp**	*5’gaactgacgaacatcattg3’*	*5’gctggcattagcaacacctg3’*

To determine the presence of *P. gingivalis* in the hippocampus, we performed an immunofluorescence assay to visualize neurons and the bacteria. Fixing and pre-treatments were performed as described above, but here, samples were incubated overnight at 4°C with PBS-TX containing primary antibodies anti-tubIII (1:300, Abcam, ab18207) and anti-RgpA1 (1:200, RgpA R1, Biorbyt, UK). Sections were washed 4-times for 5 min in PBS and incubated for 2 h with secondary fluorescent antibodies (Alexa Fluor^®^ 488 1:300 or Alexa Fluor^®^ 633 1:300). The Hoechst reagent (1:10,000, Sigma, St Louis, MI, USA) was used for nuclear staining. Endogenous tissue background immunofluorescence control was performed by the direct observation of the hippocampal tissue under the confocal microscope. Also, a primary antibody control staining was performed to discard any background that could interfere with the analysis of RgpA1 staining. A z-stack of 1.5 μm sections was captured from the CA1 hippocampal region in a confocal microscope (Nikon C2+, Melville, NY). Fluorescence intensity was measured with the confocal microscope NIS-Elements software viewer 4.0 and with ImageJ free viewer software.

### Data Analysis

The OASIS maze data were expressed as the mean ± SD of distance (cm) or time (s) units. Cytokine production was expressed as the mean ± SD of concentration (pg/ml). Alveolar bone resorption was represented in terms of distance units (nm) and expressed as the mean ± SD. The Shapiro-Wilk test was used to determine the normal distribution of the data, which were analyzed using the ANOVA-Tukey or Kruskal Wallis-Dunn tests using the SPSS v.15.0 software (IBM, NY, USA). Differences were considered as statistically significant when *p*-*value <*0.05.

## Results

### Bone Resorption Quantification

**Figure 1:**
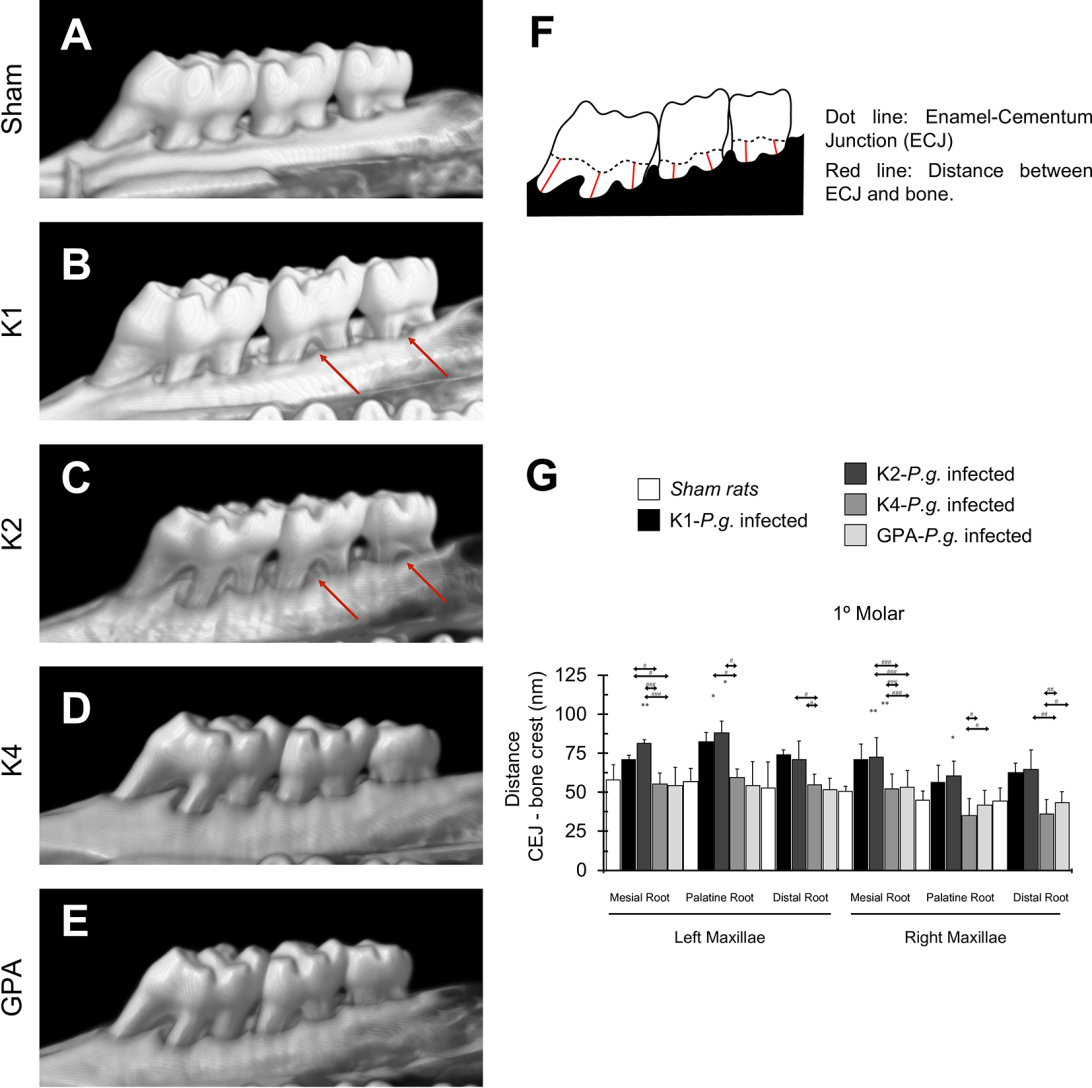
Bone resorption in the 1st molar. **(A)** micro-CT reconstruction of the left and right maxillae of sham rats. **(B–E)** micro-CT reconstruction of maxillae of rats inoculated with strains K1, K2, K4, or GPA of *Porphyromonas gingivalis.* Red arrows indicate macroscopic changes in the alveolar bone, highlighting the presence of furcation, which exists when periodontal disease has caused resorption of bone. **(F)** Scheme representing the measures design for bone resorption quantification. **(G)** Bone resorption quantification of 1st molar. Data, mean ± SD, are represented as distance (nm) for six independent experiments. *p < 0.05, **p < 0.01, compared to the control group; ^#^ *p* < 0.05, ^##^ *p* < 0.01, ^###^ *p* < 0.001, compared to the group indicated.

In order to determine whether the inoculation of *P. gingivalis* induces bone resorption, each maxilla was reconstructed in 3D images (**Figures 1A–E**). Three-dimensional quantification of alveolar bone loss was performed by determining the CEJ-ABC distances (**Figure 1F**). *P. gingivalis*-infected rats with capsular serotypes K1 or K2 displayed significantly increased alveolar bone loss compared to both *P. gingivalis*-infected rats with K4, GPA, and sham rats, as indicated by an increase in CEJ-bone crest distance of each root in the palatal root of 1st molar (**Figure 1G**). These results were also consistent when 2^nd^ and 3^rd^ molars (**Supplementary Figure 1**) were analyzed.

### Spatial Memory and Learning

**Figure 2:**
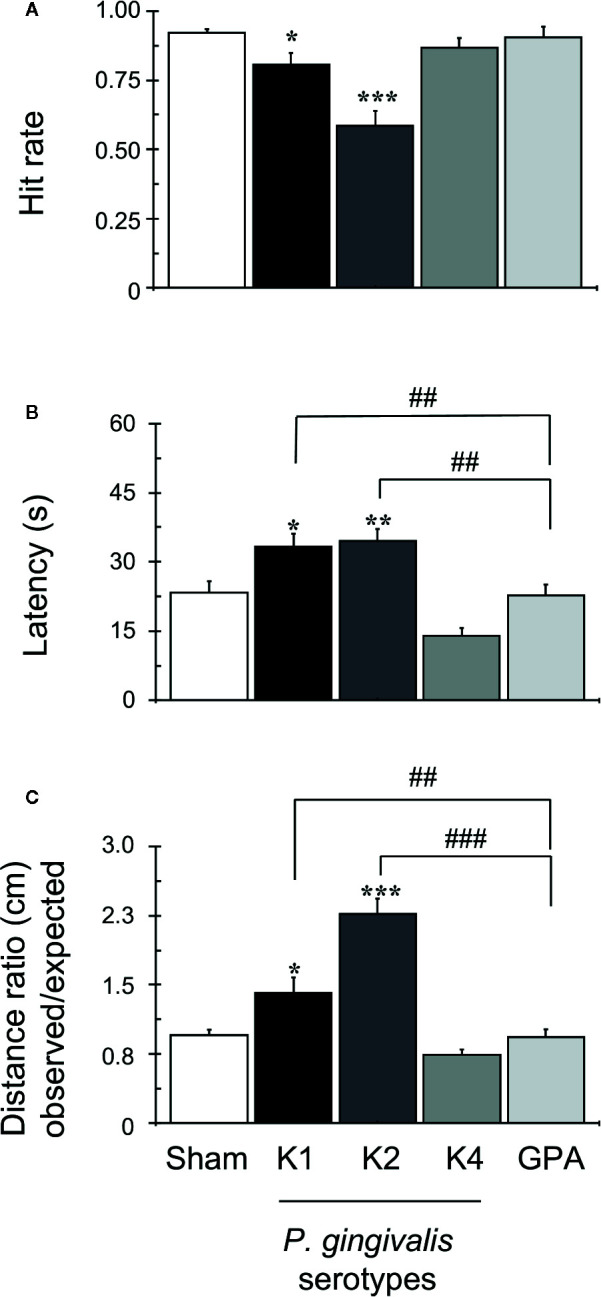
OASIS Maze task. The graph corresponds to the performance of all groups on day 6 of the task, as indicated. Each bar represents the mean ± SD of 90 videos: 15 videos were collected for each animal, out of a total of six animals per group. **(A)** Hit rate achieved during training in OASIS Maze by rats infected with *P. gingivalis*, which represents the percent of the times that the animal obtained the reward over the total trials per day. **(B)** Latency, defined as the total time (s) rats take to find the reward and complete the task, represented as mean ± SD. **(C)** The distance ratio represents the ratio between the observed/expected distances walked by rats during the task, represented as mean ± SD. *p < 0.05, **p < 0.01, ***p < 0.001, compared to the control group; ^##^p < 0.01, ###p < 0.001, compared to the group indicated.

Along the daily training sessions composed of 15 trials, since the 5^th^ day of training rats infected with capsular-serotypes K1 or K2 exhibited worse spatial memory than animals infected with K4, GPA, or sham (**Supplementary Figure 2**). On the 6^th^ day (last day of the task), sham rats and those infected with K4 or GPA displayed significantly higher hit rates, which are defined as the number of times the rat found the reward over the 15 trials, compared to rats infected with K1 or K2 capsular serotypes (**Figure 2A**). Likewise, the average latency to find the reward was significantly lower in the control groups and K4 or GPA infected rats, when compared to K1 or K2 infected-rats (**Figure 2B**). Finally, the ratio of the observed over the expected (shortest) distance covered by the animals while searching for the reward was higher in the *P. gingivalis* K1 or K2 injected group (**Figure 2C**) than that displayed by the sham group or by rats injected with K4 or GPA.

### Cytokine Production

**Figure 3:**
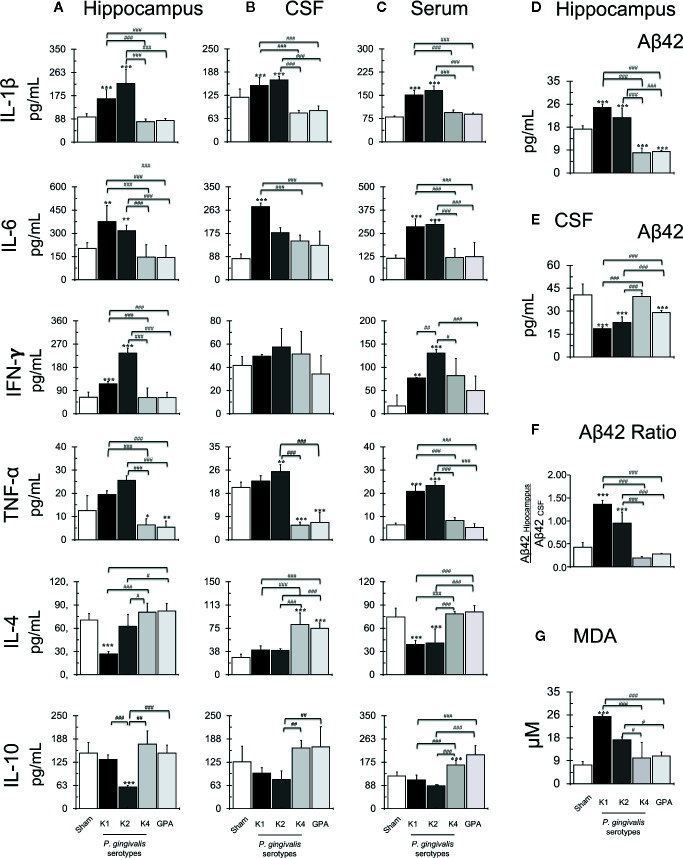
Quantification of secretion levels of pro-inflammatory molecules. Secretion levels of IL-1β, IL-4, IL-6, IL-10, TNF-α, and IFN-γ determined in the hippocampus **(A)**, serum **(B)** or CSF **(C)** from sham and *P. gingivalis* infected-rats. Data are presented as cytokine concentration (pg/ml); mean ± SD of 4 independent experiments. **(B)** Secretion levels of Aβ_1-42_ peptide on the hippocampus **(D)**, CSF **(E)** and ratio **(F)** from sham or *P. gingivalis* infected-rats. Data are presented as molecule concentration (pg/ml); mean ± SD for 4 independent experiments. **(D)** Secretion levels of Aβ_1-42_ peptide on CSF from sham or *P. gingivalis* infected-rats. Data are presented as molecule concentration (pg/ml); mean ± SD for 4 independent experiments. **(E)** The ratio of the secretion levels of Aβ_1-42_ on the hippocampus and CSF from sham rats or *P. gingivalis* infected-rats. Production levels of MDA **(G)** on the hippocampus from sham or *P. gingivalis* infected-rats. Data are presented as molecule concentration (pg/ml); mean ± SD for four independent experiments. Each experiment was performed in duplicate. **p < 0.01, ***p < 0.001, compared to the control group; ^#^ *p* < 0.05, ^##^ *p* < 0.01, ^###^ *p* < 0.001, compared to the group indicated. IL, interleukin, TNF, tumor necrosis factor, IFN, interferon, CSF, cerebrospinal fluid.

We quantified the secretion levels of the cytokines IL-1β, IL-4, IL-6, IL-10, TNF-α, and IFN-γ production in the hippocampus, CSF and serum by ELISA (**Figure 3A–C**, respectively), the levels of extracellular Aβ_42_ peptide on the hippocampus and CSF (**Figures 3C, D**) and calculated the Aβ_42_ hippocampus/CSF ratio (**Figure 3E**). Statistically significant increased production of IL-1β was detected in the hippocampus, CSF, and serum of rats infected with the capsular-serotypes K1 or K2 compared to sham, which exhibited IL-1β concentration values similar to those observed in rats infected with the capsular-serotypes K4 or GPA. IL-6 levels were also significantly increased in the hippocampus and serum of rats infected with the capsular-serotypes K1 or K2 compared to sham, which exhibited no differences in samples from rats infected with the capsular-serotypes K4 or GPA. However, in CSF, only in samples from rats infected with the capsular-serotype K1 a significant increase in IL-6 levels in comparison to all the other conditions was observed. IFN-γ levels were significantly increased in the hippocampus and serum of rats infected with the capsular-serotypes K1 or K2 compared to sham, which exhibited IL-1β concentration values similar to those observed in rats infected with the capsular-serotypes K4 or GPA. Of note, the effect observed in serum from rats infected with the capsular-serotype K2 was significantly higher than in serum obtained from rats infected with the capsular-serotype K1. However, no differences in the IFN-γ levels were detected in the CSF for all these experimental conditions. Also, increased production of TNF-α was detected in serum from K1 or K2 infected-rats compared with sham, K4 or GPA injected rats. In the CSF, the increase in the concentration of TNF-α was only observed in samples from rats injected with the capsular-serotype K2.

Interestingly, the injection of the K1-isogenic non-encapsulated mutant GPA and the K4 serotype *P. gingivalis* strain resulted in a statistically significant decrease in the levels of TNF-α. Also, significantly increased IL-4 and IL-10 secretion levels were detected in the hippocampus and CSF from rats infected with K4 or GPA, compared with those infected with K1 or K2. Also, in rats inoculated with K1 or K2 serotypes, a statistically significant increase in Aβ42 hippocampus/CSF ratio was detected, compared with the other conditions. The K1-isogenic non-encapsulated mutant and the K4 serotype *P. gingivalis* strain injections resulted in a significant decrease in Aβ_42_ levels in the hippocampus. Still, this difference was not significant when the Aβ_42_ hippocampus/CSF ratio was calculated in comparison to the sham infected control. Finally, to determine if all these stressful changes induce lipid peroxidation, we quantified MDA production in the hippocampus of periodontitis rat models (**Figure 3F, G**). An increase in MDA levels was detected in the hippocampus from K1 and K2 infected rats compared with sham, K4, or GPA infected rats.

### Glial Activation and Tau Protein Phosphorylation

**Figure 4:**
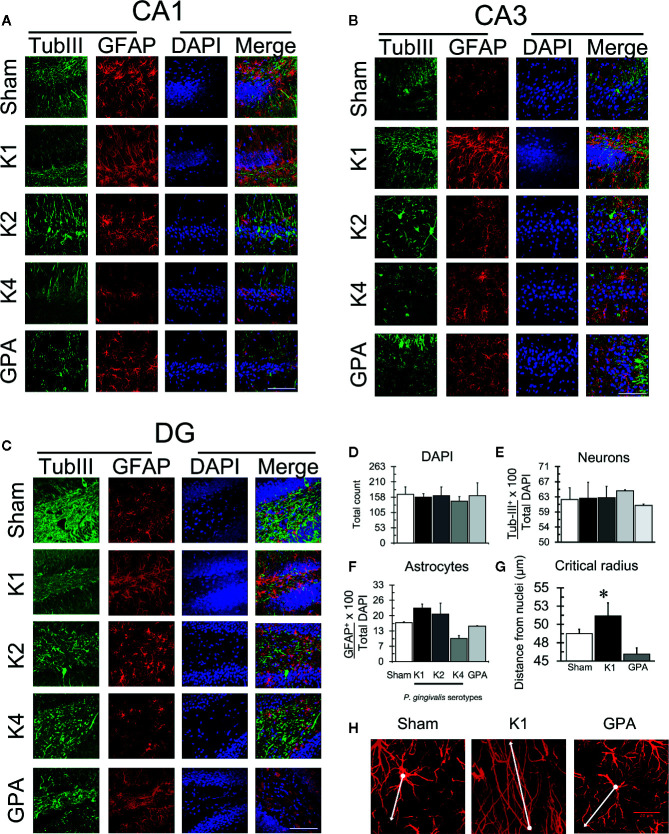
Astrogliosis detection by immunofluorescence. Representative confocal microscopy images of a 36-slices 3D projection of **(A)** CA1, **(B)** CA3, and **(C)** DG hippocampal regions from sham or *P. gingivalis* infected-rats, fixed after the sixth training session and labeled with glial fibrillary acidic protein (GFAP)- antibody (green), neuronal-specific cytoskeleton protein tubulin III (Tub-III)—antibody (red) and DAPI for the nucleus (blue). A merged image of the triple-staining is also shown. Scale Bar: 100 µm. **(D)** Total DAPI quantification. **(E)** Total neuron quantification. **(F)** Total astrocyte quantification. **(G)** Sholl analysis of the critical radius of astrocytes. **(H)** Representative confocal images show the determination of the critical radius of astrocytes from K1 *P. gingivalis*-infected rats versus sham or GPA infected rats. The white dot identifies the nuclei and the dash the most extensive prolongation. A white line joins dot and dash. Each image corresponds to a 36-slice 3D projection. Scale Bar: 20 µm. The primary monoclonal antibodies used were: anti-tubIII (1:300, ab18207), and anti-GFAP (1:300, ab10062). The secondary monoclonal antibodies were conjugated with Alexa Fluor^®^488 1:300 or Alexa Fluor^®^633 1:300. *p < 0.05.

**Figure 5:**
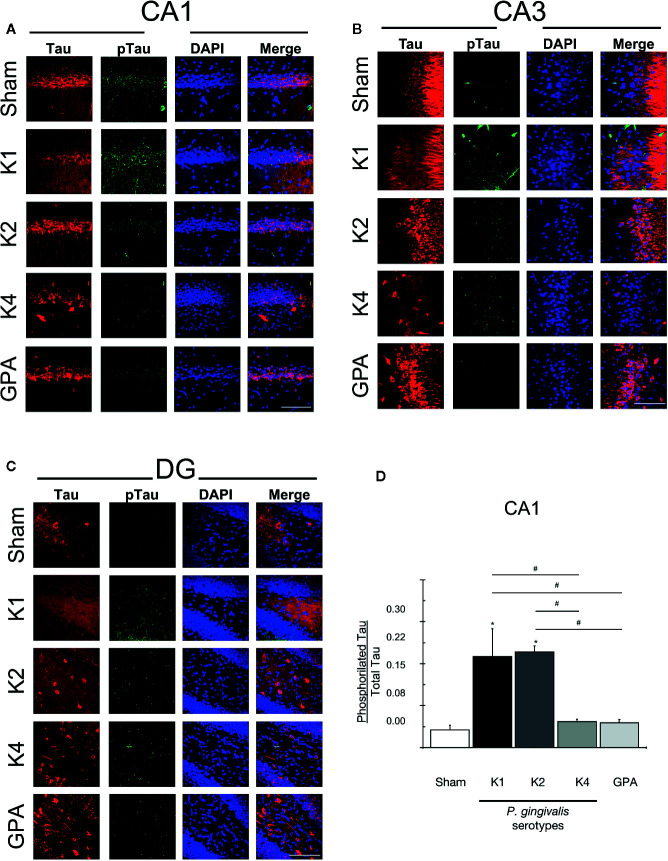
Tau and phosphorylated Tau detection by immunofluorescence. Representative confocal microscopy 36-sliceS 3D projection images of **(A)** CA1, **(B)** CA3, and **(C)** DG hippocampal regions from sham and *P. gingivalis* infected-rats, fixed after the sixth training session and labeled with anti-Tau (red), anti-phosphorylated (phospho Tau) antibody (green) and DAPI for the nucleus (blue). A merged image of the triple-staining is also shown. Scale Bar: 100 µm. **(D)** pTau/Tau ratio. An increase in pTau stain was observed in rats infected with encapsulated serotypes K1 or K2 compared with the other conditions in the CA1 region. *p < 0.05, compared to control group; ^#^ *p* < 0.05, compared to the group indicated. The primary monoclonal antibodies used were: anti-Tau (1:300, ab64193), and anti-phospho-Tau (1:300, Phospho S404, ab64193). The secondary monoclonal antibodies were conjugated with Alexa Fluor^®^488, 1:300, or Alexa Fluor^®^633 1:300.

After the Oasis maze task, rat hippocampal samples were evaluated for the occurrence of astrogliosis and phosphorylation of Tau protein by immunohistochemistry (**Figures 4**, **5**). For this purpose, we visualized neurons and astrocytes in the CA1, CA3, and DG regions of the hippocampus (**Figures 4A–C**) and quantified the total number of cells in the CA1 region by counting DAPI-stained nuclei, the number of TubIII-positive and GFAP-positive cells for all experimental groups (**Figures 4D–G**). We observed no differences in the total number of astrocytes and neurons when comparing all the conditions tested. However, we noted that astrocytes of rats infected with the K1 or K2 serotype exhibited an unusual, distinctive morphology in the CA1, CA3, and DG regions. We thus performed a Sholl analysis to compare the morphology of these cells in the CA1 region, in the hippocampus of sham, K1 or GPA infected rats and observed that K1 infection promoted a slight increase in the size and the complexity of astrocytic processes (**Figure 4H** and **Supplementary Figure 3**).

Finally, to further search for AD-markers in the brains of orally-infected rats, we performed immunohistochemistry to stain Tau protein and its phosphorylated-form (pTau) and quantified the pTau/Tau ratio, which increase is considered a characteristic feature of the disease (**Figures 5A-C**). An increase in pTau staining was observed in the CA1 hippocampal region from rats infected with the serotypes K1 or K2 compared with sham rats and with K4 or GPA infected rats (**Figure 5D**).

### Detection of *P. gingivalis*

**Figure 6:**
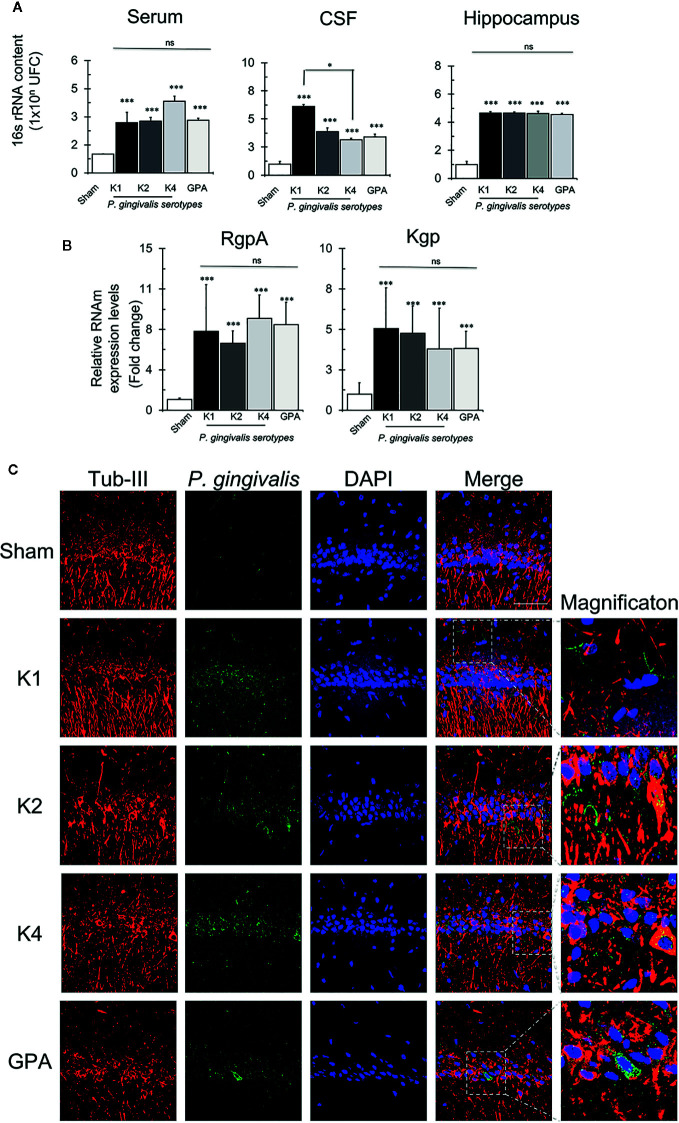
P. gingivalis detection by qPCR and immunofluorescence. **(A)** Absolute quantification of the 16S subunit of *P. gingivalis* in serum, CSF and hippocampus of sham- or rats infected with the serotype K1, K2, K4, or GPA. Results expresses as mean± SEM of four independent rats in each condition. CFU, colony-forming units, CSF, cerebrospinal fluid. *p < 0.05. **(B)** Detection of RgpA and Kgp genes of *P. gingivalis* in the hippocampus. Relative expression levels of RgpA and Kgp of *P. gingivalis* in hippocampi of sham rats infected with the serotype K1, K2, K4, or GPA ***p < 0.001. Results expresses as mean± SEM of four independent rats in each condition **(C)** Immunofluorescence detection of *P. gingivalis*. Immunofluorescence of the CA1 region of the hippocampus of Sprague-Dawley rats for the detection of gingipain R1 (RgpA). In red, neurons labeled with Tubulin III), in green *P. gingivalis* labeled with RgpA, in blue the nuclei (DAPI) and in merge. The superposition of the three channels. 2D images obtained from the 35-slice 3D projection taken from the two hemispheres from two independent rats in each condition. Scale Bar: 20 μm. ns, non significative.

Further, to investigate the potential spreading capacity of *P. gingivalis*, we set to detect the presence of the bacteria in serum, cerebrospinal fluid (CSF), and hippocampus. To this aim, we measured the bacterial load by quantifying the 16S rRNA subunit of *P. gingivalis* by qPCR (**Figure 6A**). We found that after 55 days of the first inoculation of 1 x 10^10^ CFU/ml in palatal mucosa, the different serotypes were detectable in serum, CSF and Hippocampus. In serum, we found 1 x 10^3^ CFU/ml, in CSF 1 x 10^4^ CFU/ml and in hippocampus, 1 x 10^5^ CFU/ml for all bacterial serotypes, but no significant differences were found among the different conditions in hippocampus and serum. However, in CSF, K1 was increased compared to K4 serotype. We quantified also RgpA and Kgp gingipain genes in hippocampal tissue by qPCR (**Figure 6B**). We found that both genes were detected in hippocampus, but no differences were found among the different serotypes. Finally, to visualize the localization of the bacteria in hippocampal tissue, the gingipain RgpA component of *P. gingivalis* was immunodetected in the CA1 region of the hippocampi collected from rats exposed to all the experimental and control conditions (**Figure 6C**). Immunodetection of RgpA in the CA1 region indicated its co-localization with different cell types, including neuronal cells, for all the experimental conditions.

## Discussion

Different studies have reported that the presence of chronic inflammatory disease, such as periodontitis, would increase inflammatory mediators, anaerobic bacteria, or virulence factors in the systemic circulation. Chronic oral infection of mice with *P. gingivalis* results in a specific immune response, significant increases in oral bone resorption, aortic inflammation, and viable bacteria in oral epithelium and aorta, allowing constant exposure of periodontal bacteria to systemic circulation (45). According to the infectious origin of neuroinflammation, bacteria, virulence factors, and systemic pro-inflammatory mediators could spread to the central nervous system through the systemic circulation or brain nerves (27). Also, it is proposed that different microorganisms would use the Aβ peptide as a substrate or source of adhesion (46). Once in the brain, these agents can induce microglial pro-inflammatory cytokine secretion, characterized by significant increases in the levels of IL-1β, IL-6, TNF-α, and IFN-γ, which induce a reactive-astrocyte phenotype (29). Reactive astrocytes express increased levels of the three components necessary for Aβ production: APP, β-secretase, and γ-secretase, the enzymes that jointly cleave APP to produce Aβ. Reactive astrocytes secrete 7-fold more Aβ_42_ than neurons, and the Aβ_42_ is 60% truncated by non-enzymatic modifications, which induce its hydrophobicity and synaptotoxicity (47). This chronic inflammatory response in the brain would thus increase Aβ secretion and/or decrease its clearance causing its accumulation in the brain (48, 49). An increase in the concentration or accumulation of Aβ_42_ facilitates peptide aggregation in oligomeric and fibrillar forms linked to synaptotoxicity. As a consequence, the intracellular cytoskeleton-associated protein suffers abnormal Tau hyperphosphorylation, which in turn induces loss of neuronal function (50), and these sequential events would lead to neuronal death and spatial memory and learning deficits (26, 51).

Several studies have analyzed the relationship between periodontitis and AD and established that *P. gingivalis* cause cognitive impairment and induce brain pro-inflammatory cytokine release (21–25, 32, 34, 45, 52). From them, some studies used transgenic mice (21, 23, 25, 32, 45) and other studies used wild-type animal models (22–24, 32, 52). Only one of these studies reported a direct association between periodontitis-related alveolar bone resorption and deficits in learning and memory (21). For periodontitis induction, some studies used oral bacterial irrigation or oral gavage, (21, 23, 32, 52) two studies intraperitoneal injections of LPS, (22, 24), and two studies direct inoculation of LPS or lysed bacteria to the brain (25, 34). This technical variability to induce periodontitis makes it challenging to compare the reported results. Although all these studies demonstrate, to a greater or lesser extent, that cognitive deterioration is associated with periodontitis, it remains to be elucidated whether these effects are a consequence of some periodontitis molecular mediator, the direct activity of the bacteria, general sepsis, or alteration of the intestinal microbiota, based on direct interaction between periodontal and gut microbiota. Accordingly, to identify the role of a bacterium or virulence factor in periodontitis, the model we chose was the palatal inoculation, which has been previously used by our research group in mice and known by causing an increased Th1/Th17 immune response, osteoclast activity, and alveolar bone resorption (10, 44).

LPS constitutes an important virulence factor for *P. gingivalis*, being composed of lipid A, a central core of oligosaccharides and O-polysaccharide or O-antigen (O-Ag) (53). For lipid A, five isoforms have been described: 2 tetra-acylated and 3 penta-acylated, which induce different immune responses (54, 55). Although lipid A has potential immunogenicity, these effects have not been observed in humans, suggesting that human immune cells are not able to recognize the structural differences of lipid A, in the same way as animal immune cells do (55–57). One reason for this might reside in the fact that *P. gingivalis*-secreted gingipains degrade the CD14 receptor, the primary lipid A receptor in macrophages (58–61). In addition to lipid A, structural variability has also been described in the O-antigen, but its role in the pathogenicity remains unclear (62). All *P. gingivalis* strains possess LPS. Unfortunately, the studies that induced periodontal infection using LPS to evaluate the consequences in the brain, do not specify the strain of origin of the LPS used (22, 24, 25, 63). As above mentioned, it is relevant to know the origin of the LPS, since the variability of Lipid A of the LPS of *P. gingivalis* is important when analyzing the immunogenicity of this antigen, which is not necessarily associated with the pathogenicity of *P. gingivalis.* Lipid A can be tetra or penta-acylated. If it is tetra-acylated, it is immunogenic and is capable of inducing a CD14-mediated response in immune cells, but penta-acylated LPS does not induce an immune response (54, 55, 57, 62, 64). In general, clinical isolates of *P. gingivalis* from healthy subjects have a modified LPS with substitutions and lack O-antigen (65, 66) and also, their main receptor, the CD14 molecule, is a substrate of gingipains (58–60).

Besides LPS, gingipains and capsular polysaccharides are central in the mechanisms of pathogenesis of *P. gingivalis* infection. *P. gingivalis* is the only bacterium that produces gingipains, which are the endopeptidases responsible for 85% of its proteolytic activity (4, 5). They can be detected and isolated in aggregates with hemagglutinin, hemoglobin or other gingipains, or attached to the outer membrane of *P. gingivalis* (67, 68). In general terms, gingipains are essential for *P. gingivalis* growth, attachment and migration, and also for fimbria maturation (5, 69, 70). The gingipain *knock-out* is not compatible with bacterial life (71). Interestingly, gingipains degrade the receptors that recognize LPS in the host cells, and this degradation is considered a mechanism of evasion of the immune response (58–60). It was recently reported that gingipain inhibition reduces the bacterial load of an established *P. gingivalis* brain infection, decreases neuroinflammation, and rescues neurons in the hippocampus, revealing that this endopeptidase has a pivotal role in the mechanism of brain damage induced by *P. gingivalis* (34). Gingipains are likely to participate in the process of neuronal tissue invasion (38).

Without detriment to other virulence factors that *P. gingivalis* possesses, the capsular polysaccharides or K-antigens, which constitute the main macromolecule of its surface, are responsible for its serotypification, defining the taxonomic classification and contributing to the virulence (72, 73). The immunogenic role of *P. gingivalis *capsule has been previously demonstrated, and the structural variability of its polysaccharide component has been directly associated with its virulence potential (6–10, 74). Several encapsulated and non-encapsulated (K1-K7) serotypes of *P. gingivalis* have been described; among them, the encapsulated serotypes display more virulence in experimental infections (10, 72, 75, 76). Also, a *P. gingivalis* K1-isogenic non-encapsulated *knock-out* mutant ΔPG0116-PG0120 (GPA) has been developed, allowing to test the effects of the absence of capsule (14).

Given that there is differential microbiological susceptibility to periodontitis, which is determined by the presence of strains belonging to different serotypes in the oral cavity, it is reasonable to postulate that this susceptibility could also be observable in brain cells. Therefore, we decided to investigate the potential deleterious effects of each serotype of *P. gingivalis* in the brain. In the present study we investigated, for the first time, the effects of palatal injections of different serotypes of *P. gingivalis* on rat spatial memory and hippocampal histopathology. To this aim, animals received two injections separated by an interval of 1 week, into the palatal mucosa of 10^10^ CFU/ml of the strains W50 (serotype K1), HG184 (K2), or ATCC^®^ 49417™ (K4) of *P. gingivalis*. Control animals were inoculated with the non-encapsulated W50 ΔPG0116-PG0120 mutant strain (GPA) of *P. gingivalis*. Making a parallel with the normal daily bacterial load from chronic periodontal disease, these values in the humans depends on the patient’s diagnosis: in healthy subjects the burden of *P. gingivalis* ranges from 6 x 10^2^ to 8.6 x 10^4^ CFU/ml. In patients with stage III periodontitis, it can vary from 4 x 10^6^ to 9 x 10^5^, or even 1 x 10^12^CFU/ml. However, as the severity of the disease increases, the burden of *P. gingivali*s decreases (77, 78).

Our study ratified the higher capacity of the serotypes K1 and K2 of *P. gingivalis* to induce alveolar bone resorption during experimental periodontitis. Besides, we report for the first time that periodontal infections induced with strains K1, K2, K4, or GPA of *P. gingivalis* differentially affected the rat brain. The presence of *P. gingivalis* was determined by quantifying the total bacterial load in serum and hippocampus. Although the identification of the bacteria in the serum has been previously reported (10), the presence of all *P. gingivalis* serotypes in the brain is the most intriguing and novel finding of our work. After 55 days, all *P. gingivalis* strains were detected in both samples, demonstrating that it is possible to detect *P. gingivalis* in the brain, regardless of the capsule and its virulence. The explanation to the fact that even the GPA strain entered the brain may reside on fimbria. Fimbria is the most extensive portion of the virulence factors of *P. gingivalis*, which allows it to adhere to any surface, its maturation is dependent on gingipains and is one of the migration mechanisms (5, 69, 79, 80). Indeed, fimbria allows *P. gingivalis* to invade the cells or tissues of the host and even the W50 (K1) strain, in the absence of the long fimbria, maintains its adherent capacity (81–83). In mutant strains of gingipains, *P. gingivalis* loses its ability to adhere and migrate, but in mutant capsule strains, LPS, gingipains and fimbria are not affected, evidenced in the maintenance of the adherence and migration capacity of these strains (10, 14, 84, 85).

Our results indicate that despite all the strains can achieve the brain, only the most virulent strains of *P. gingivalis*, K1 and K2, effectively induced pro-inflammatory cytokine production, astrogliosis, Aβ_42_ secretion, Tau hyperphosphorylation, and cognitive decline in young rats, after a short period of infection exposure. Since our experimental model consists of wild type rats injected with *P. gingivalis* and examined for the presence of brain Aβ amyloid 55 days after bacterial injection, we did not expect yet to detect the presence of accumulated Aβ in plaques. Nevertheless, our protocol did detect monomeric forms as well as soluble oligomeric conformations of Aβ, which are pivotal to trigger Tau hyperphosphorylation, and neuronal dysfunction, leading to cognitive deficits in AD pathology (39, 86–91). We show that periodontal infections with the encapsulated serotypes K1 or K2 of *P. gingivalis* induced neuroinflammation, astrogliosis, cognitive decline, and histopathological signs of AD in the hippocampus, as compared with the less virulent K4 strain and GPA. It worth noting that, instead, K4 and GPA strains promoted the increase in the secretion levels of immunomodulatory cytokines, such as IL-4 and IL-10, and that astrogliosis was considerably reduced in these rats. Unlike in several of the previous studies, (21, 22, 24, 34, 52) we carried out periodontal infections using inoculation of complete bacteria, with their full antigenic potential, which is considered the standard protocol that allows determining the local and systemic effect of a single bacterium (92). We show that, in contrast to the deleterious effects triggered by the serotypes K1 and K2 on the brain, periodontal infections induced with strains K4 or GPA provoked no significant changes in comparison to sham-controls. The present work is the first study to demonstrate the role of the encapsulated and non-encapsulated strains of *P. gingivalis* in the pathogenesis of AD and the differential capacity to induce neuroinflammation in the hippocampus, with the concomitant spatial memory loss. This study corroborates and adds to recent findings showing that *P. gingivalis* infection impairs cognition in wild-type or transgenic mice for amyloid precursor protein (APP), Cathepsin-β, or ApoE^-^/^-^ (21, 22, 24, 25, 32, 34, 52). However, we further show that only the pathogenic serotypes of the bacteria selectively activate the inflammatory response in the brain.

Compared to previous reports utilizing the entire *P. gingivalis*, our study present novel findings, since all of these works used ATCC^®^33277™ or FDC 38 *P. gingivalis* strains, which are less virulent strains. Some of them have been unable to capture all AD hallmark features like Tau hyperphosphorylation as we did. One of these studies compared the effects of ATCC^®^33277™ strain in young and middle-age rats, and showed that *P. gingivalis* oral infection induced memory impairment and neuroinflammation only in the middle-age mice, suggesting that the effect of the bacteria is age-dependent. In contrast, using a short-term infection in young rats, we were able to find all the above-mentioned AD-features simultaneously in wild type rats, when they were orally infected with the most virulent, encapasulated K1 and K2 *P. gingivalis* serotypes. These results indicate that despite the fact that all serotypes were capable of invading the brain, their capsular K antigens were the fundamental determinants of the host response and vulnerability. Of note, our results may also shed light on previous studies in *postmortem* brains, in which the presence of the *P. gingivalis* 16S rRNA gene was detected not only in the AD brains but also in five of the six nondemented control brains examined. The PCR analysis of *P. gingivalis* in the *postmortem* brains and CSF reported by these authors did not differentiate between *P. gingivalis* strains (34). Therefore, the possibility that some strains might be more virulent than others in causing Alzheimer´s disease and that healthy brains exhibit less virulent strains should be evaluated in the future.

One of the limitations of our study is that our experimental model, which can be considered a short-term inflammatory condition, is rather like an infectious state in humans, equivalent to a condition such as sepsis. The provision of anti-inflammatory medication would be really useful to demonstrate whether the cognitive function is a short-term effect of the pro-inflammatory state or a long-term degenerative change. Also, considering that periodontitis is a multibacterial disease, it would be interesting to contemplate the virulence effects of the other periodontal and gastrointestinal bacteria to which the human body is exposed to on a daily basis. In this regard, *Aggregatibacter actinomycetemcomitans* is recognized to be highly virulent; studies are in progress in our research group to determine whether this bacterium can also generate neuroinflammation *in vivo*, since we previously reported that serotype b of *Aggregatibacter actinomycetemcomitans* triggers pro-inflammatory responses and amyloid beta secretion in hippocampal cell cultures (37).

## Conclusions

Periodontitis is a major risk factor for Alzheimer´s disease, and *P. gingivalis* is a keystone pathogen linking these diseases. Although different *P. gingivalis* serotypes can access the brain, its particular capsular types play a central role in the chronic inflammatory assault and cognitive impairment induced by short-term oral infection. The more virulent encapsulated serotypes are likely more prompt to lead to AD-like-pathology, probably speeding up the pathogenic process compared to less virulent serotypes.

## Data Availability Statement

The raw data supporting the conclusions of this article will be made available by the authors, without undue reservation.

## Ethics Statement

The experimental protocols were approved by the Bioethics Committees on Animal Research of the Faculty of Medicine (protocol #CBA 0755 FMUCH) and the Faculty of Dentistry (protocol #17085-ODO-UCH), Universidad de Chile.

## Author Contributions

JD-Z and AP-L substantially contributed to conception and design of the experiments, acquisition, analysis, and interpretation of data and they drafted and critically revised the manuscript. JM, JV and RV contributed to conception and design of the experiments, acquisition of data and they also critically revised the manuscript. SM-R, MJ-U, FV-O, CM-M, and GM contributed to acquisition of data and critically revised the manuscript. All authors contributed to the article and approved the submitted version.

## Funding

This research was financed by grants (FONDECYT 1150736, FONDECYT 1181780) from the Chilean Governmental Agencia Nacional de Investigación y Desarrollo (ANID), by ICM P-09-015, by a grant (FIOUCh 17/019) from the Faculty of Dentistry and by the Regional Development Program of the International Association for Dental Research (RDP-IADR).

## Conflict of Interest

The authors declare that the research was conducted in the absence of any commercial or financial relationships that could be construed as a potential conflict of interest.
