# Supplementary material for: Alzheimer’s Disease-Like Pathology Triggered by Porphyromonas gingivalis in Wild Type Rats Is Serotype Dependent
Source: Front Immunol. 2020 Nov 9;11:588036. doi: 10.3389/fimmu.2020.588036 (PMC7680957; doi:10.3389/fimmu.2020.588036)
Supplement: Supplementary file 1 [file Presentation_1.pptx]

## Slide 1
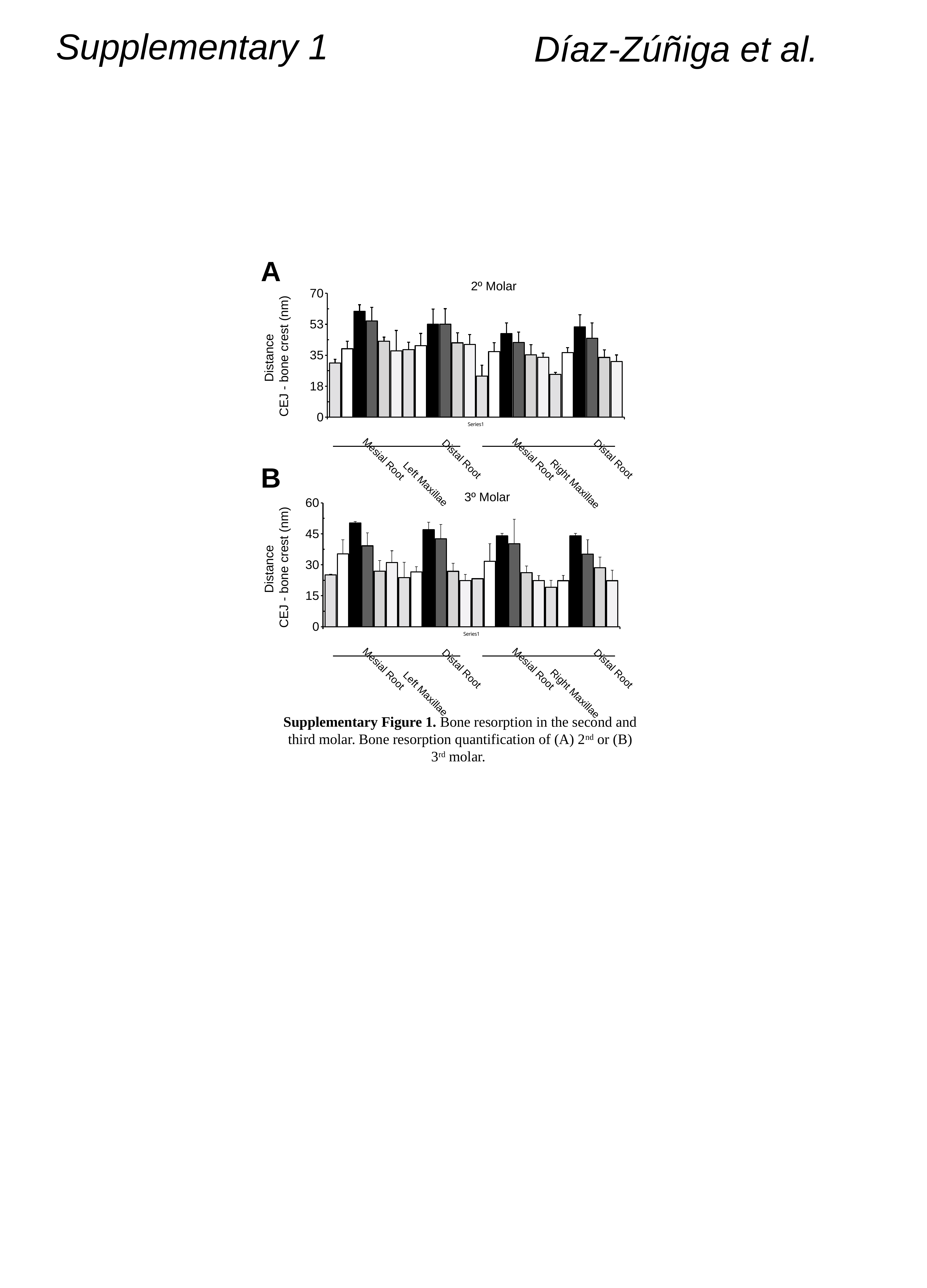

Supplementary 1
Díaz-Zúñiga et al.
A
2º Molar
[unsupported chart]
Distance
CEJ - bone crest (nm)
Mesial Root
Distal Root
Mesial Root
Distal Root
Left Maxillae
Right Maxillae
B
3º Molar
[unsupported chart]
Distance
CEJ - bone crest (nm)
Mesial Root
Distal Root
Mesial Root
Distal Root
Left Maxillae
Right Maxillae
Supplementary Figure 1. Bone resorption in the second and third molar. Bone resorption quantification of (A) 2nd or (B) 3rd molar.

## Slide 2
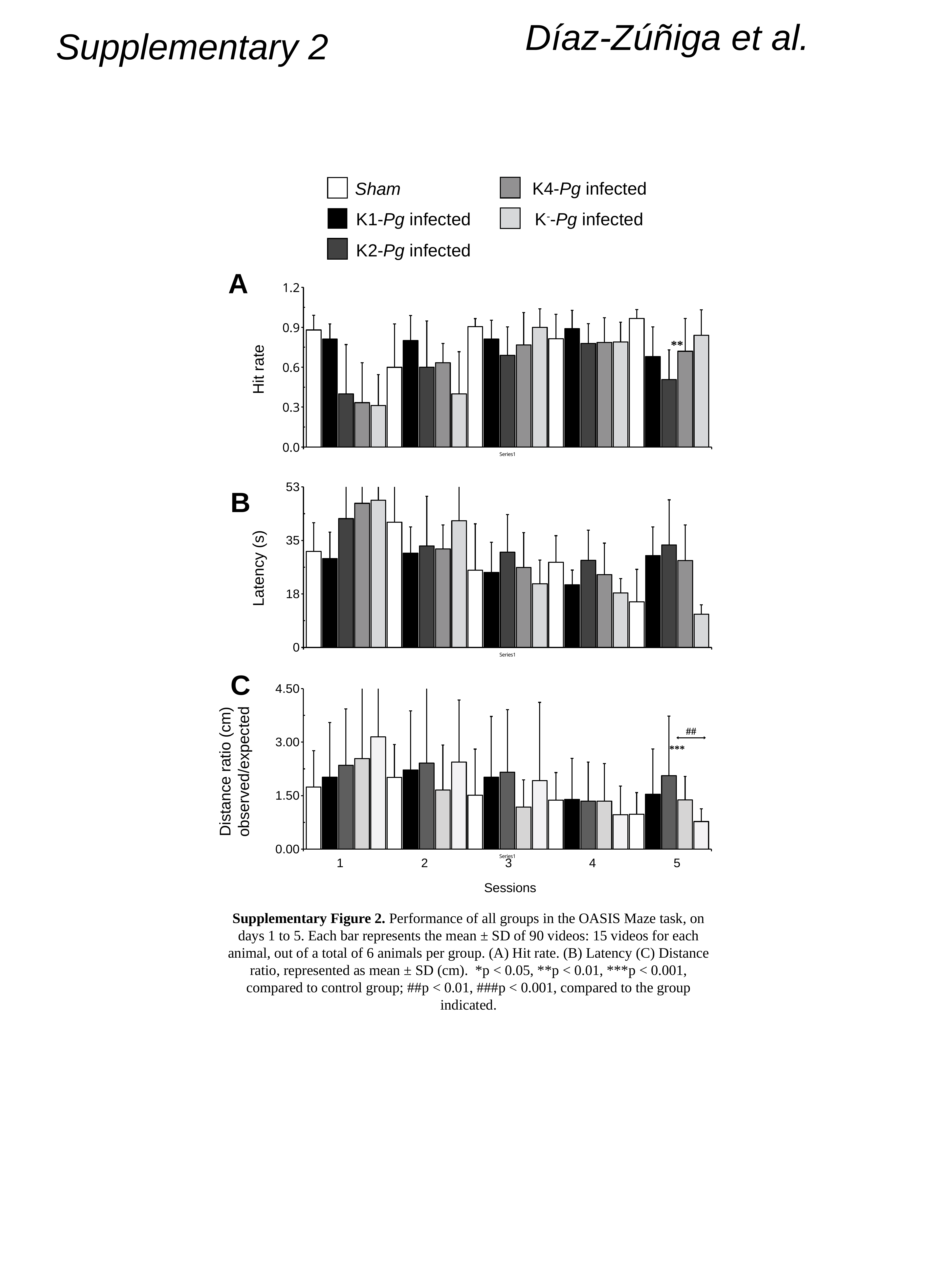

Díaz-Zúñiga et al.
Supplementary 2
K4-Pg infected
Sham
 K--Pg infected
K1-Pg infected
K2-Pg infected
A
[unsupported chart]
**
Hit rate
[unsupported chart]
B
Latency (s)
C
[unsupported chart]
##
***
Distance ratio (cm)
observed/expected
1
2
3
4
5
Sessions
Supplementary Figure 2. Performance of all groups in the OASIS Maze task, on days 1 to 5. Each bar represents the mean ± SD of 90 videos: 15 videos for each animal, out of a total of 6 animals per group. (A) Hit rate. (B) Latency (C) Distance ratio, represented as mean ± SD (cm). *p < 0.05, **p < 0.01, ***p < 0.001, compared to control group; ##p < 0.01, ###p < 0.001, compared to the group indicated.

## Slide 3
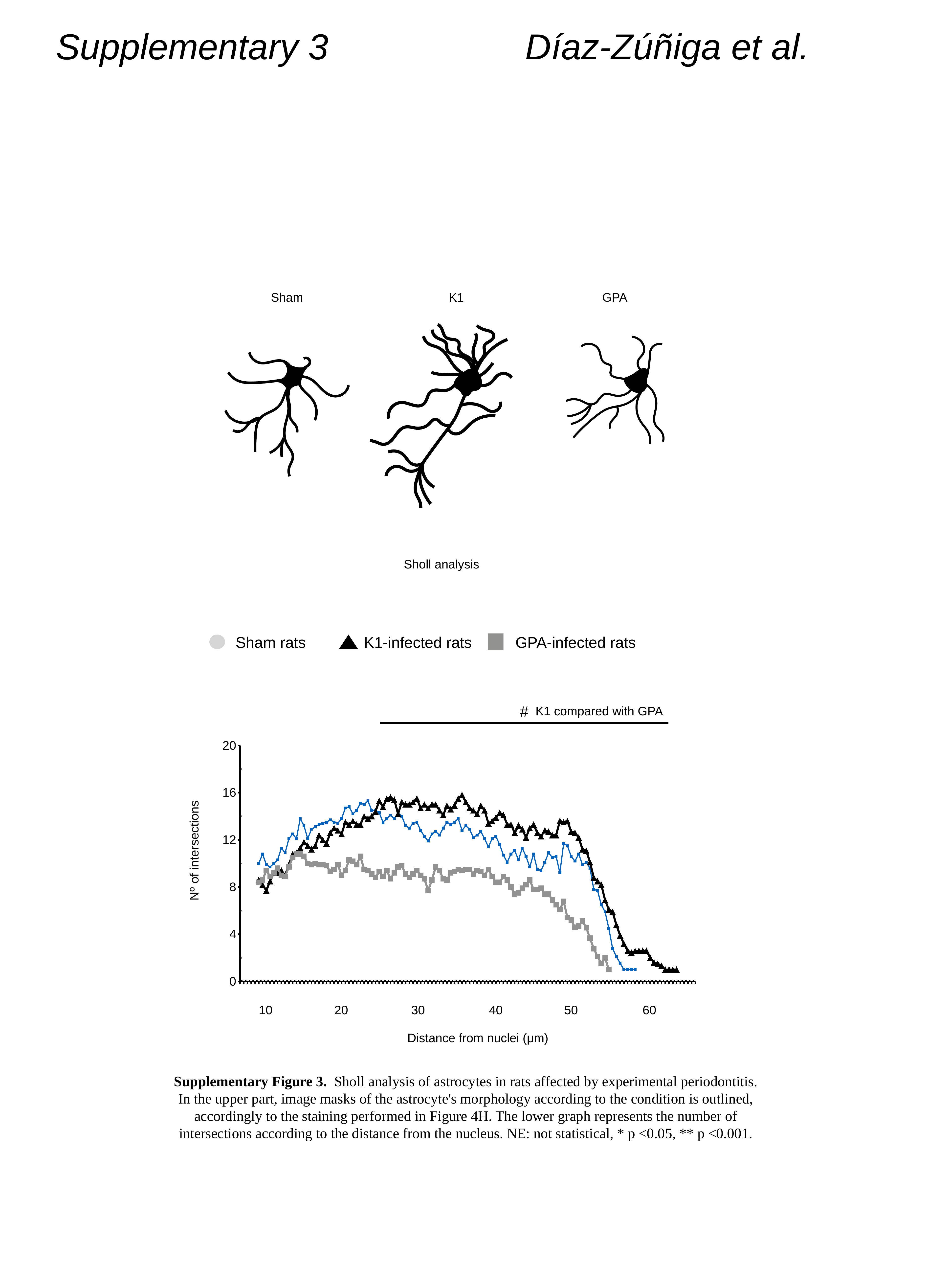

Supplementary 3
Díaz-Zúñiga et al.
Sham
K1
GPA
Sholl analysis
Sham rats
K1-infected rats
GPA-infected rats
K1 compared with GPA
#
[unsupported chart]
Nº of intersections
50
60
10
20
30
40
Distance from nuclei (μm)
Supplementary Figure 3. Sholl analysis of astrocytes in rats affected by experimental periodontitis. In the upper part, image masks of the astrocyte's morphology according to the condition is outlined, accordingly to the staining performed in Figure 4H. The lower graph represents the number of intersections according to the distance from the nucleus. NE: not statistical, * p <0.05, ** p <0.001.
